# Fear of COVID-19 and Life Satisfaction: The Role of the Health-Related Hardiness and Sense of Coherence

**DOI:** 10.3389/fpsyt.2021.712103

**Published:** 2021-11-01

**Authors:** Joanna Dymecka, Rafał Gerymski, Anna Machnik-Czerwik, Romuald Derbis, Mariola Bidzan

**Affiliations:** ^1^Department of Health Psychology and Quality of Life, Institute of Psychology, Opole University, Opole, Poland; ^2^Department of General and Work Psychology, Institute of Psychology, Opole University, Opole, Poland; ^3^Department of Clinical and Health Psychology, Institute of Psychology, University of Gdańsk, Gdańsk, Poland

**Keywords:** fear of COVID-19, life satisfaction, sense of coherence, hardiness, mediation

## Abstract

**Introduction:** The COVID-19 pandemic is contributing to increased fear and anxiety throughout society, which may affect life satisfaction. Health-related hardiness and sense of coherence (SOC) are personal resources that help people adapt to difficult circumstances. The purpose of this study was to investigate the relationship between fear of COVID-19, SOC, health-related hardiness, and life satisfaction.

**Methods:** A total of 907 Polish people (522 women and 385 men) participated in this study. The Fear of COVID-19 Scale (FOC-6), the Health-Related Hardiness Scale, the Sense of Coherence Scale (SOC-29), and the Satisfaction with Life Scale were used.

**Results:** Correlation showed that fear of COVID-19 was negatively related to health-related hardiness, SOC, and life satisfaction. Health-related hardiness and SOC were positively related to life satisfaction. Both SOC and hardiness were mediators between fear of COVID-19 and life satisfaction during the current pandemic.

**Conclusion:** SOC and health-related hardiness are personal resources that are important for dealing with the effects of the COVID-19 pandemic. According to our study, SOC and hardiness can mediate between fear of COVID-19 and life satisfaction. Presented cross-sectional results have to be verified in future longitudinal studies in order to strengthen the conclusions presented in this manuscript. This study verified the role of only two personal resources, so more research is needed on the role of other personal resources during COVID-19 pandemic.

## Introduction

For over a year, the entire world has been struggling with a global pandemic caused by the spread of the SARS-CoV-2 coronavirus, which appeared in the city of Wuhan, Hubei province, China in late 2019. In early 2020, the disease caused by the virus was called COVID-19 and, on March 11, 2020, the WHO declared it a global pandemic. It is the largest pandemic to affect humans so far in the twenty first century. The clinical course of the disease varies from mild or even asymptomatic to severe respiratory failure and death. The prognosis is worse in the elderly and patients with comorbidities (1–3). According to the World Health Organization (4), more than 126 million people in the world have been infected and more than 2.8 million have died. The first case of COVID-19 in Poland was recorded on March 4, 2020, and by March 28, 2021, almost 2.2 million people were infected and more than 51,000 have died.

COVID-19, like other contagious diseases which cause epidemics, affects not only physical health but also mental functioning. The changes in everyday life caused by the pandemic were rapid and unprecedented. COVID-19, as a global threat to public health, requires drastic control measures and has disrupted almost every aspect of everyday life. The rapid increase in confirmed cases and deaths, isolation, reduced social contact, school and workplace closures, and significant restrictions on activity and freedoms can all be stressful for society as a whole (5, 6). Even if most people are not infected and remain physically well, they often suffer from the negative psychological effects of the epidemic.

An infectious disease pandemic can also affect life satisfaction, which is an individual's cognitive evaluation of their life (7). Many previous studies have shown that experiencing difficult life situations has a negative impact on human health and well-being (8). Several studies have shown that the pandemic affects well-being and life satisfaction (6, 9–11). A Turkish study showed that fear of COVID-19 decreases life satisfaction (12), while Harper et al. (10) indicated that fear of COVID-19 reduces individuals' well-being.

A contagious disease pandemic increases fear and anxiety throughout society, as can be observed in both the current and previous epidemics (13). During the current COVID-19 pandemic, people fear becoming infected, dying or losing loved ones, and contact with people who might be infected (6, 14, 15). Fear is an adaptive protective mechanism for animals and humans that is fundamental to survival and involves several biological processes related to preparing to respond to potentially dangerous events. However, when it is chronic or disproportionate, it can cause mental disorders (16). Ahorsu et al. (17) indicated that fear of COVID-19 increases levels of psychological distress and has a negative impact on mental health. Constant information about the many fatalities around the world and the growing number of cases lead to increased fear of COVID-19, causing people to experience stress, anxiety, and mood disorders, which have a negative impact on their psychological well-being (12, 18). Coronavirus threatens one's safety and desire to survive, which affects quality of life. Therefore, fear of COVID-19 reduces people's well-being and decreases their life satisfaction (19, 20). However, Özmen et al. (19) showed that fear of COVID-19 explains only a small percentage of the variation in life satisfaction.

Hardiness is usually defined as a generalized style of functioning characterized by a high level of commitment, control, and challenge, thanks to which the negative effects of stress are mitigated. People with a high level of hardiness believe that they have control over their life and that through their commitment to their goals they will achieve positive results. They treat everyday stressors as challenges (21). The research showed that people characterized by a high level of hardiness were protected against the negative impact of stress on their life and health (22, 23).

Sense of coherence (SOC) is defined as “a global orientation that expresses the extent to which one has a pervasive, enduring though dynamic feeling of confidence that (1) the stimuli deriving from one's internal and external environments in the course of living are structured, predictable and explicable; (2) the resources are available to one to meet the demands posed by these stimuli; and (3) these demands are challenges, worthy of investment and engagement” (24).

Antonovsky (24) in his works drew attention to the relationship between SOC and hardiness. Both postulate the existence of complex personality traits that act as personal resources in stressful situations (24, 25). Also, the mechanisms by which SOC and hardiness affect physical and mental health seem to be similar (26). Therefore, many studies conducted on various populations indicate a relationship between SOC and psychological hardiness (26–28).

SOC seems to be a particularly important resource for dealing with the pandemic. Many studies have shown that high levels of SOC make it easier to accept inevitable difficulties (29, 30). Also, SOC is particularly necessary when strong stressors affect an individual (31), as in the case in the ongoing pandemic. Studies have also shown that a strong SOC is negatively associated with anxiety, perceived stress, and its consequences (32). People with a strong SOC can more effectively deal with adverse circumstances (33). SOC and hardiness are also resources that affect quality of life. The role of SOC as a significant predictor of quality of life has been demonstrated in many previous studies and analyses that found that higher SOC is linked with better quality of life (34–36). Also, hardiness can improve individuals' well-being and increase life satisfaction (37). Many studies on different populations have shown that hardiness is positively related to quality of life (28, 38) and life satisfaction (39).

Several studies conducted during the COVID-19 pandemic have shown that hardiness is a very important resource for dealing with adverse events stemming from COVID-19 (40, 41). Hardiness enables us to perform and to stay focused during hard times. It helps people to adapt to new situations and withstand adversity (21). Hardiness plays a protective role by reducing the risk of dysfunctional stress reactions occurring in emergency workers during the current COVID-19 pandemic (42). Importantly, it has also been shown that hardiness is positively related to individuals' well-being during the current pandemic (43).

In addition, many studies on different populations have shown that SOC and hardiness act as mediators in the relationships between a variety of variables (29, 30, 44–48). SOC has been found to mediate the relationship between adverse experiences and psychological well-being (29), and to mediate between symptoms, stress, coping, and life satisfaction (49). Hardiness has been found to be a mediator between stress and illness (50). Hardiness has been found to act as a mediator of the relationship between traumatic experiences and post-traumatic adaptation (51). Therefore, it can be assumed that SOC and hardiness will be mediators of the relationship between fear of COVID-19 and life satisfaction during the global pandemic.

### The Present Study

The purpose of this study was to investigate the relationship between fear of COVID-19, SOC, health-related hardiness, and life satisfaction. Based on the information presented in the introduction, we decided to investigate whether: (1) fear of COVID-19 is significantly and negatively associated with SOC and hardiness; (2) SOC and hardiness are significantly and positively associated with life satisfaction; and (3) SOC and hardiness are mediators in the relationship between fear of COVID-19 and life satisfaction.

## Materials and Methods

### Participants

A total of 907 Polish people (522 women and 385 men) participated in this study. The average age of all respondents was 39.28 years. Above half of the study participants had university level education. The second largest group were people with high school education. The smallest number of respondents had elementary and vocational education. About half of the studied sample represented people living in towns. The obtained number of people living in cities and villages was relatively equal. About 70% of the participants in this study were employed. We didn't verify the reasons of unemployment of the rest of the participants. The level of other sociodemographic variables has not been investigated. Detailed sociodemographic data of the studied sample were presented in Table 1.

**Table 1:** Characteristics of the studied sample (*N* = 907).

	***M***	***SD***	***Min***	***Max***
**Age**				
All participants	39.28	15.30	18.00	102.00
Women	38.59	15.22	18.00	101.00
Men	40.21	15.38	18.00	102.00
	***N***		**%**	
**Gender**				
Women	522		57.55%	
Men	385		42.45%	
**Education**				
Elementary School	28		3.09%	
Vocational	65		7.17%	
High School	347		38.26%	
University	467		51.49%	
**Place of Residence**				
Village	220		24.26%	
Town	410		45.20%	
City	277		30.54%	
**Professional Activity**				
Unemployed	268		29.55%	
Employed	639		70.45%	
	***N***		**%**	
Population (15/03/2021; Polish Central Statistical Office)	38° 070 317		100%	
Studied sample (% as a part of whole Polish population)	907		<0.01%	

### Procedure

The recruitment of the study participants was carried out using the snowball method. Due to the pandemic, every effort was made to ensure that the study was completely safe for its participants. Therefore, we recruited respondents via the Internet. The recruitment of the participants took place between March and May of 2020. Study assistants were asked to share the survey on social media platforms. All people under 18 years of age were excluded from the analysis. Digitally excluded older adults were able to complete the survey by phone (*n* = 11). The study participants were informed about the anonymity of the study. They could stop filling out the survey at any time and without giving any reason. All respondents gave informed consent to participate in this study. The presented project adhered to the guidelines of the Bioethics Committee of the University of Opole.

### Measures

Fear of the coronavirus was measured with the Fear of COVID-19 Scale (FOC-6) (6). Respondents answered the questions using a five-point scale (1— “definitely disagree”; 5— “completely agree”). Higher FOC-6 scores indicate higher fear of COVID-19. FOC-6 is a reliable questionnaire (Cronbach's *alpha* = 0.83; McDonald's total *omega* = 0.84). Confirmatory Factor Analysis indicates that the obtained model fit values are mostly acceptable (*CFI* = 0.957; *TLI* = 0.928; *RMSEA* = 0.111). After setting the error covariance between items 1 and 2 (which is theoretically justified due to the semantic similarity of those items), FOC-6 model fit coefficients improve (*CFI* = 0.984, *TLI* = 0.969, *RMSEA* = 0.072). Confirmatory Factor Analysis (CFA) results show acceptable model fit coefficient values for the tested 6-items one-factor model. Unfortunately, FOC-6 wasn't developed with the usage of the Exploratory Factor Analysis (EFA). Although the results of the *post-hoc* EFA based on Oblimin rotation with the usage of Maximum Likelihood extraction method confirm the results of the CFA analysis (one-factor model where all factor loadings exceed the value of 0.60), it cannot be certain that the structure would be the same as if EFA had been used during the questionnaire validation process. What is more, FOC-6 validity wasn't verified with relation to other fear of COVID-19 related scales, but it can be supported by the fact, that it's results are significantly related to other similar variables, such as stress or well-being measures (6).

The Sense of Coherence Scale (SOC-29) (52), adapted by Koniarek et al. (53), was also used in this study. The questionnaire consists of 29 items related to various aspects of human life. The study participants responded to them on a seven-point scale. The questionnaire is used to study global SOC and its three components: the senses of comprehensibility, manageability, and meaningfulness. Only the global score was used in this study. The scale shows good reliability (in this study Cronbach's α was 0.91). Higher SOC-29 scores indicate higher SOC.

Hardiness was measured with the short version of the Health-Related Hardiness Scale (HRHS) by Pollock (54), in its Polish adaptation by Dymecka et al. (28). It contains 12 items that participants assess on a six-point Likert scale, where 1 indicates complete disagreement and 6 indicates complete agreement. The scale shows good reliability (in this study Cronbach's α was 0.78). Higher HRHS scores indicate higher hardiness.

The Satisfaction with Life Scale (SWLS) (7), adapted by Juczyński (55), was also used. It consists of five questions on a seven-point scale (1— “definitely disagree”; 4— “neither agree nor disagree”; 7— “completely agree”). The scale shows good reliability (in this study Cronbach's α was 0.87). Higher SWLS scores indicate higher life satisfaction.

### Statistical Analysis

In order to verify the formulated hypotheses, a number of statistical analyses were used. The significance of the relationship between fear of COVID-19, hardiness, SOC and life satisfaction was tested with Pearson's *r* correlation analysis. It allowed us to verify the two-sided relationships between tested variables. Before analyzing the mediational role of hardiness and SOC in the relationship between fear of COVID-19 and life satisfaction, we decided to verify if the residuals autocorrelation and multicollinearity between hardiness and SOC may have occurred. Therefore, Durbin-Watson test and Variable Inflation Factors analysis were used. One-sided relationships and indirect effects of the possible mediators were verified with mediation analysis using MODEL 4 of PROCESS v3.4 macro (56). Additionally, we decided to perform a *post-hoc* power analysis in order to check whether the obtained sample allows conclusions from the presented data. For that purpose, we used the Monte Carlo simulation (57) performed in the R environment (58). A significance level of α = 0.05 was adopted as the threshold value for statistical significance in all analyses, which is a standard practice in the presented field of research. Bootstrapping mediation using the PROCESS macro was performed with 5,000 samples (59).

## Results

### Correlation Analysis

In the first step of the statistical analysis, it was decided to check whether there were any significant relationships between the tested variables. In order to select an appropriate analysis, it was checked whether the distributions of the examined variables showed large asymmetry. Skewness and kurtosis statistics showed that the studied distributions did not show large asymmetry. On this basis, a parametric Pearson's *r* correlation analysis was performed. Pearson's *r* correlation showed significant relationship between fear of COVID-19, health-related hardiness, SOC, and life satisfaction. Relationship between fear of COVID-19 and other variables was negative and weak. What is more, health-related hardiness was positively and moderately related to SOC, and life satisfaction. More detailed data are shown in Table 2.

**Table 2:** Results of Pearson's r correlation analysis (*N* = 907).

	***M***	***SD***	***SKE***	***K***	**1**.	**2**.	**3**.	**4**.
1.Fear of COVID-19	21.41	5.84	−0.39	−0.69	–			
2.Health-related hardiness	51.76	9.14	−0.25	−0.14	−0.17^***^	–		
3.Sense of coherence	131.24	24.12	−0.10	0.07	−0.14^***^	0.41^***^	–	
4.Life satisfaction	22.67	5.99	−0.41	−0.25	−0.11^**^	0.20^***^	0.61^***^	–

** *p < 0.01;*

*** *p < 0.001; SKE, skewness; K, kurtosis*.

### Mediation Analysis

In the next step, it was decided to perform a mediation analyses due to the significant relationships found using the Pearson's *r* correlation analysis. Before calculating the mediation analyses, it was decided to verify if the tested residuals are correlated. Durbin-Watson test results showed, that the residuals were not correlated in the verified model. Based on the small asymmetry of the studied distributions and the lack of autocorrelation, it was decided to perform the mediation analysis as planned. Two separate MODEL 4 mediation analyses were performed, because the analysis of *Variable Inflation Factors* suggested that multicollinearity between hardiness and SOC may have occurred (VIF > 10).

First, it was examined whether hardness mediates the relationship between fear of COVID-19 and life satisfaction. The second model tested whether SOC was a mediator in the relationship between fear of COVID-19 and life satisfaction. The PROCESS macro results showed that all investigated mediation paths were statistically significant. Analysis of the confidence intervals of the indirect effects suggests, that health-related hardiness and SOC mediated the relationship between stress and life satisfaction. More detailed data is shown in Table 3.

**Table 3:** Detailed data of the results of the PROCESS MODEL 4 analysis (*N* = 907).

**Mediator: Health-related hardiness**									
**Path**			**Symbol**	***Beta***	***b***	***SE***	***p***	***LLCI***	***ULCI***
*X*	–>	*M_1_*	a_1_	−0.17	−0.26	0.05	<0.001	−0.364	−0.163
*M_1_*	–>	*Y*	b_1_	0.18	0.12	0.02	<0.001	0.074	0.159
*X* (*M_1_*)	–>	*Y*	c'	−0.07	−0.07	0.03	0.029	−0.140	−0.007
Indirect Effect of *M_1_*			a_1_* b_1_	−0.03	−0.03	0.01	–	−0.049	−0.016
**Mediator: Sense of Coherence**									
*X*	–>	*M_1_*	a_2_	−0.14	−0.57	0.13	<0.001	−0.836	−0.302
*M_2_*	–>	*Y*	b_2_	0.15	0.61	0.01	<0.001	0.138	0.164
*X* (*M_2_*)	–>	*Y*	c'	−0.02	−0.02	0.03	0.453	−0.074	0.033
Indirect Effect of *M_2_*			a_2_* b_2_	−0.08	−0.09	0.02	–	−0.129	−0.045
			***DW***	***p***	**Autocorrelation**				
Durbin-Watson test			1.94	0.358	0.03				

*X, Fear of COVID-19; M_1_, Health-Related Hardiness; M_2_, Sense of Coherence; Y, Life Satisfaction; LLCI, Lower Level Confidence Interval; ULCI, Upper Level Confidence Interval; DW, Durbin-Watson' test statistic*.

### Power Analysis

Monte Carlo simulation (57) with 5,000 replications and 20,000 Monte Carlo draws was performed at the confidence level of 95%. The simulation confirmed the power obtained at the level of at least 0.98 for both mediation models.

## Discussion

This study aimed to determine the relationship between fear of COVID-19, SOC, health-related hardiness, and life satisfaction during the coronavirus pandemic. Presented results show that fear of coronavirus was negatively linked to life satisfaction of the studied sample. Our results are consistent with previous studies, which show that pandemic situation can negatively affect our life satisfaction (6, 9–11). There are multiple possible explanations of this significant result. In the COVID-19 pandemic, fear of infection, death, and loss of loved ones are common, which leads to increased distrust of others, avoidance, and withdrawal from everyday activities (60). At the beginning of the pandemic, we all had to adapt to new living conditions, which could produce a sense of uncertainty related to the development of the epidemiological situation. An increase of anxiety during a pandemic is a natural reaction. It can cause high levels of stress, which has a negative impact on our well-being. Even though our study did not verify the role of social support, it is possible that pandemic opportunities for interpersonal contact, resulting in disruption of social support networks at the time when they may be most needed (61), because, as is known, social support is crucial for adaptive functioning (15, 62).

The current study showed also a negative correlation between fear of COVID-19 and two personal resources: health-related hardiness and SOC. In line with theoretical assumptions, the present study found a correlation between SOC and psychological hardiness. Both SOC and hardiness were mediators between fear of COVID-19 and life satisfaction during the current pandemic. According to theory, hardiness can affect perceptions of stressful events (63). In the current pandemic situation, people with high levels of hardiness may be confident that they will be able to protect themselves from infection or, in the event of infection, be able to deal with it effectively (64). It can therefore be assumed that they will cope better with the fear of COVID-19. The tension caused by a pandemic may not turn into distress and they will be less likely to experience its negative consequences, such as anxiety or depression. Thanks to this, despite difficult circumstances, they might judge their life as satisfactory. It is possible because hardy people adapt more easily to difficult life situations such as the pandemic. Also, people with high levels of hardiness become engaged in what they do, don't feel alienated, usually believe that they can at least partially control what happens to them, do not feel powerless, and treat changes as challenges and opportunities for development, rather than as threats (65). Hardiness is related to the tendency to perceive stressful life events as less serious, less dangerous, and more manageable (21, 66).

SOC can play an equally important role in the process of dealing with the COVID-19 pandemic. SOC can, by effectively managing stress and reducing levels of anxiety, affect psychological well-being and quality of life (67, 68). In his salutogenic theory, Antonovsky (24) repeatedly emphasized the role of SOC in an individual's coping with difficult situations, because, in his opinion, SOC reduces the likelihood of strong tension turning into stress, which is extremely important during a pandemic. A person with a strong SOC sees the world as orderly and understandable, and finds order in the environment, helping them to better cope with chaotic stimuli. A strong SOC can allow a person to approach difficult situations as challenges rather than obstacles. A high SOC is also associated with an appropriate response to emotional stimuli, with low sensitivity to them and high emotional resilience. People with strong SOC seek information only when they need it to solve a problem, and not when it causes overload (24), which is particularly important during the current pandemic, as excessive focus on negative information provided in the media can lead to increased anxiety (69). In a situation where the problem cannot be solved, people with a high SOC can adapt better and thus suffer less. Another important role of SOC is to influence the emotions experienced in difficult situations. SOC may limit the experience of negative emotions in stressful situations, which is particularly important in dealing with fear of COVID-19. People with a strong SOC experience emotions consciously and they can provide a motivational basis for action (70). Therefore, the SOC's mediational role between fear of COVID-19 and life satisfaction seems theoretically justified and was empirically verified in the presented study.

The results obtained in the present study were also confirmed in previous studies. Research suggests that constant information about confirmed deaths and the growing number of cases increased levels of fear of COVID-19, which had a negative impact on life satisfaction (12). Health-related fear, decreased availability of social support, and the curtailment of typical recreational activities have diminished well-being and life satisfaction throughout society (71). The link between the fear of COVID-19 and hardiness was also confirmed in previous studies. In Russian research, it was shown that low levels of hardiness were associated with high levels of fear of COVID-19 (72). A negative relationship between hardiness and negative emotions such anxiety and depression has already been demonstrated in many previous studies (73–78). Studies conducted during the COVID-19 pandemic have shown that psychological hardiness is negatively correlated with anxiety, depression, and the general severity of psychopathological symptoms. Therefore, we believe that hardiness can be associated with changes in anxiety levels during the pandemic. People with lower hardiness show increased anxiety over time (71). Studies have shown that the lower the hardiness level, the greater the assessment of the negative aspects of COVID-19. For people with low levels of hardiness, the pandemic may be a source of stress that affects their quality of life (72), which is a possible explanation of the presented results.

In many studies on various populations, it has been found that SOC was negatively correlated with levels of fear, stress, and anxiety (67, 79). In previous studies, SOC was associated with lower emotional tension and lower levels of situational anxiety (70). The relationship between SOC and hardiness has also been documented in other empirical works (26–28, 80). Studies suggest that psychological hardiness is an important resource for coping with the COVID-19 threat (41). One Russian study found hardiness to be a personal adaptive resource in stressful situations related to the COVID-19 pandemic (71). We believe that people with high levels of hardiness might interpret stressful life events as being less difficult (21). Hardiness contributes to perceiving the pandemic as a challenge. High levels of hardiness can help a person control anxiety and irrational thoughts. This resource prevents unpleasant emotions and thoughts, which have a negative effect on various stress factors and secondary trauma (42).

Studies have shown that people with high hardiness have better quality of life and are more energetic and optimistic (81). In studies among elderly people, it has been shown that higher levels of psychological hardiness are associated with greater life satisfaction (39). Hardiness protects one's well-being in the face of negative life events (21). It prevents the deterioration of psychological well-being in stressful situations (71). This is why research has shown that psychological hardiness is positively related to well-being during the COVID-19 pandemic (43). This was also confirmed in our study, which showed a positive relationship between hardiness and life satisfaction during the COVID-19 pandemic.

The relationship between SOC and life satisfaction has been demonstrated in many empirical studies (82–85). Data from empirical studies confirm that SOC can affect life satisfaction, acceptance of inevitable difficulties, and sense of control of situations. Many empirical studies have confirmed the relationship between SOC and subjective well-being, quality of life, and satisfaction with life (83). SOC helps one perceive the disease as less threatening (86). A strong SOC is particularly important when an individual experiences very difficult situations (31, 87), and the pandemic is undoubtedly one such situation. Many studies have also confirmed the role of SOC as an important mediator of the relationship between a variety of variables, including life satisfaction, as demonstrated in the present study. SOC has been shown to be a mediator between stress and life satisfaction during the COVID-19 pandemic (82). Another study on found that the relationship between adversity—indicated by the occurrence of worry, anxiety, and stress—and life satisfaction can be explained by the significant mediation of SOC (29). SOC can act as a protective factor in the process of adaptation to difficult life events (49), and the mediation path found in this study can help us understand how it does so. Other study results indicate that SOC can explain more variance in many areas of quality of life than any other variable (88). Therefore, we believe that this might be a possible explanation why SOC played as a significant mediator in the relationship between fear of COVID-19 and life satisfaction.

Although our study produced important results, it is not free of limitations. Although our mediation model is tested as causal, it does not allow us to establish cause and effect relationships, since our data are cross-sectional. It would require longitudinal studies to confirm whether the proposed direction of the influence is correct. Additionally, recruiting study participants via social media is an example of convenience sampling. Such sample does not have sufficient power to detect sociodemographic group differences, which makes it limiting in the case of statistical analyses that can be used (89). Therefore, another study based on population-based sampling should be performed in order to confirm the proposed mediation model and possible group differences. Lastly, this study verified the role of fear of COVID-19 with a FOC-6 questionnaire. Although it is a reliable scale, its model fit coefficient values are acceptable at best. Additionally, FOC-6 was validated without the usage of Exploratory Factor Analysis. Next studies should be performed with a scale representing better psychometric properties, such as FCV-19S (17). FCV-19S wasn't used in the presented study due to the fact, that it started before FCV-19S was yet available. Also, the validity of FOC-6 should be verified by correlating its results with the results of the FCV-19S scale.

Previous studies have shown that SOC and health-related hardiness are personal resources that are important in the current COVID-19 pandemic. The increasing number of infections, the millions of people who have lost their lives, and the inefficiency of health care systems are leading to increased fear. Resources such as SOC and psychological hardiness can mediate the effects of fear on life satisfaction. That assumption was positively verified in the presented manuscript. Research on hardiness and SOC in COVID-19 pandemic is very important, because people with a high level of personal resources may experience greater satisfaction with life despite the duration of the pandemic. For these people, fear might be less paralyzing, and they may view the pandemic situation as a challenge rather than a burden. Both resources facilitate dealing with difficulties by changing the way one relates and reacts to events outside one's control. The idea is not to try to change or divert attention from the problem of the pandemic, because the virus is a real threat and there are objective reasons to be afraid of it, but fear does not have to dominate one's existence. People with strong SOC and hardiness can function with realistic fears and relate to them in an adaptive manner (62). Therefore, when working with people experiencing the psychological consequences of the pandemic, it is worth considering their personal resources and to work on developing these resources.

## Data Availability Statement

The raw data supporting the conclusions of this article will be made available by the authors, without undue reservation.

## Ethics Statement

The studies involving human participants were reviewed and approved by University Research Ethics Committee at the University of Opole. The patients/participants provided their written informed consent to participate in this study.

## Author Contributions

JD: conceptualization, project administration, writing, and original draft preparation. RG: methodology, formal analysis, writing, and original draft preparation. AM-C: conceptualization, project administration, and writing. RD and MB: conceptualization, supervision, and investigation. All authors contributed to the article and approved the submitted version.

## Conflict of Interest

The authors declare that the research was conducted in the absence of any commercial or financial relationships that could be construed as a potential conflict of interest.

## Publisher's Note

All claims expressed in this article are solely those of the authors and do not necessarily represent those of their affiliated organizations, or those of the publisher, the editors and the reviewers. Any product that may be evaluated in this article, or claim that may be made by its manufacturer, is not guaranteed or endorsed by the publisher.
